# Dental manifestations in bariatric patients – review of literature

**DOI:** 10.1590/S1678-77572009000700002

**Published:** 2009

**Authors:** Carolina Silveira BARBOSA, Gabriel Salles BARBÉRIO, Vinicius Rizzo MARQUES, Vitor de Oliveira BALDO, Marília Afonso Rabelo BUZALAF, Ana Carolina MAGALHÃES

**Affiliations:** 1 Undergraduate student, Bauru School of Dentistry, University of São Paulo; 2 DDS, MSc, PhD, Full Professor, Department of Biological Sciences, Bauru School of Dentistry, University of São Paulo; 3 DDS, MSc, PhD, Assistant Professor, Department of Biological Sciences, Bauru School of Dentistry, University of São Paulo

**Keywords:** Bariatric patients, Dental manifestations, Gastro-esophageal reflux

## Abstract

The rate of bariatric surgery has significantly risen in the past decade as an increasing prevalence of extreme obesity can be observed. Although bariatric surgery is an effective therapeutic modality for extreme obesity, it is associated with risk factors affecting also oral health. Based on an overview of the current literature, this paper presents a summary of dental manifestations in bariatric patients. Bariatric surgeries are associated with an increased risk for gastro-esophageal reflux which in turn might account for the higher amount of carious and erosive lesions observed in bariatric patients. As a result, also dentin hypersensitivity might be observed more frequently. The current data indicate that recommended postsurgical meal patterns and gastric reflux might increase the risk for dental lesions, particularly in the presence of other risk factors, such as consumption of sweet-tasting foods and acidic beverages. Further research is needed to evaluate the correlation of bariatric surgery and the development of dental diseases.

## INTRODUCTION

Modern methods to reduce the weight of adipose patients, including bariatric surgery techniques, have been developed for the treatment of obesity especially in its morbid form^22^^,^^30^. Bariatric surgery is considered as effective and safe treatment for all ages, increasing in prevalence over the years^22^^,^^30^. Since the implementation, the surgical techniques have undergone great changes along the time, for both the improvements of the tools and the post-operative sequels^1^.

Nowadays, there are 3 main surgery techniques reported^22^. One technique is the *Gastric Band*, in which a silicone ring is placed around the stomach, thus creating two compartments: a small one above (15–20 mL) that will store small quantities of food, thus generating a sense of satiety, and the other part is larger and placed below, which will take part of normal digestion^8^. Another option is *Gastric bypass Roux-in-Y* technique, in which a small pouch is created (15 to 30 mL) stapling the stomach itself, restricting the amount of food that can be consumed. A part of the small intestine is diverted, delaying the mixing of food with gastric juice^15^. Finally, *Misuse Biliopancreatic* is performed in a way that ¾ of the stomach are removed and the intestine is shortened, reducing the time of contact of food with the intestine, considerably reducing the absorption of the nutrients^22^.

There are many factors that might influence the results. In this sense, it is worth mentioning that the elderly patients are more likely to develop post-surgical complications, attributed to lower functional reserve of this age group, in addition to the presence of other metabolic diseases such as diabetes, which led significant sequel in these patients^4^^,^^9^.

Regarding this issue, post-bariatric surgery manifestations include gastro-esophageal, respiratory, cardiovascular, endocrine and psychological changes^24^. In operated patients, the most common gastrointestinal complications found are stenosis of the duodenum, gastric ulcer, diarrhea, chronic vomiting, reflux and gastro-esophageal cancer. There is also increase risk of iron, vitamin B12, vitamin D and calcium deficiencies, mainly related to poor absorption of nutrients by the stomach and intestine^24^^,^^28^.

On the other hand, a reduction of almost 90% of cases of the asthma and the sleep apnea, related to weight loss, is reported^3^. For the cardiovascular high risk patients, decreasing in systolic and diastolic pressures with consequent reduced risk of hypertension and coronary artery disease are reported in the operated patients. Furthermore, reduction of total cholesterol, triglycerides and uric acid as well as increasing in HDL fraction of cholesterol is observed^26^. In patients submitted to gastroplastic surgery with reduction of weight, the rates of diabetes and risk for non-diabetics are reduced^5^. It is also reported changes in the level of plasmatic hormones related to ovulation, which are below normal due to alteration in gastro-intestinal absorption^25^.

Besides the physiologic factors, psychological and emotional should also be considered regarding to postoperative consequences, as these factors might influence the effects of the treatments^18^. Along with the weight loss an increase of self-esteem is observed as well as an improvement of social relationships, reduction of anxiety and depression. On the other hand, some patient may develop a self rejection, psychotic behavior, eating disorders, returning to the initial weight^24^^,^^28^. Clinical reports suggest that patients with psychiatric complications after surgery, especially with fear of returning to the previous weight, induce vomit^18^, relating at this point the psychological problem with oral manifestations.

Based on above considerations, the aim of this paper was to present a summary of dental manifestations in bariatric patients.

## REVIEW OF LITERATURE AND DISCUSSION

### Clinical evidence

Heling, et al.^14^ (2006) conducted a study with 113 patients (around 30–50 years old), who were submitted to bariatric surgery 4–5 years ago. They examined the self-assessment of bariatric patients with regard to their dental health. 79% of the patients reported vomiting as the most frequent phenomenon after surgery; 37% reported eating more sweet foods after surgery; 20% referred to improved oral hygiene; 73% did not change their habits of oral hygiene; 34% reported to have increased their visits to dentists; 60% have not changed the frequency of queries to the dentist; 37% reported major hypersensitivity after surgery; 44% reported vomiting associated with high sensitivity; 32% are suffering from indigestion after surgery and of those 59% reported hypersensitivity; and 80% of the patients have visited the dentist due to hypersensitivity. Some clinical case reports also showed the relation between bariatric surgery and increase of tooth decay^11^^,^^13^.

Bariatric surgery might affect dental health by the pH decrease caused by the high frequency of sugar ingestion as well as Gastro-esophageal reflux disease (GER). GER is a chronic condition resulting from the retrograde flow of gastroduodenal contents (mainly stomach acids, such as hydrochloric acid) to the esophagus and /or adjacent organs, such as the mouth. The pH of gastric juice is around 1.2, being a potential risk for tooth demineralization, as the critical pH for dissolution of dental apatite is around 5.5^17^. Additionally, the patients showed reduction in the production of saliva^23^, in part due to the low absorption of nutrients by the intestine, which in turn can facilitate the mineral dissolution. The reduction of pH can lead or facilitate tooth demineralization (caries and erosion) and hypersensibility^14^. The main consequences of these injuries are the enamel loss and hypersensitivity due to exposure of dentinal tubules.

### Dental Erosion

One of the lesions related to the demineralization is the dental erosion, which is defined as chemical dissolution of dental tissues by a chemical process (acid or chelating agents) without the bacterial involvement^21^. The etiology of erosion is multifactorial and not fully understood. The most important sources of acids are those found in the diet, such as acidic foods and drinks^20^ and those originated from the stomach, like gastric acids from regurgitation and reflux disorders. Currently, the increased consumption of acidic foods and soft drinks is becoming an important factor for the development of erosive wear^19^.

The acidic attack leads to an irreversible loss of dental hard tissue, which is accompanied by a progressive softening of the surface^19^. This softened zone is more susceptible to mechanical forces, such as abrasion^27^, which in turn have little or no effect on sound dental hard tissues^2^.

Clinically, early enamel erosion appears as a smooth silky-shining glazed surface. Typical for erosions of the facial aspects of teeth is a ridge of enamel that separates the defect from the marginal gingival. Occlusal erosion is characterized by rounded cusps and concavities. Further progression of occlusal erosion lead to a distinct grooving of the cusps, and restorations are rising above the level of the adjacent tooth surface. In cases of severe erosion, the whole occlusal or facial morphology disappears. When the dentin is reached, it is common report of hypersensitivity to cold, heat and osmotic pressure. Other consequences of dental erosion are diastema, thin and fractured incisal edges, loss of vertical dimension, opened pseudobite and prominence of aesthetic restorations^10^.

### Dental caries

Dental caries is a multifactorial disease, whose aetiology is related to the presence of a dental plaque composed by cariogenic bacteria, which can metabolize sugars such as sucrose. As a result of this metabolism, organic acids are produced such as lactic acid, which in turn can induce the demineralization of dental tissues^16^^,^^29^. With time, the biofilm becomes saturated regarding minerals that are released from the dental structure, favouring the precipitation and the formation of an initial subsurface carious lesion^6^^,^^12^.

The early sign of enamel lesion is characterized as white spot (known also as non-cavitated lesion) as consequence of subsurface demineralization. With time and the increase of bacterial metabolism, the intact surface layer can break down leading to formation of cavity, the spread of bacteria and progress of the lesion to dentin. Following exposure of the dentin to the masses of bacteria in the cavity, the most superficial part of dentin will soon be decomposed through the action of acids and proteolytic enzymes. This zone is referred to as the zone of destruction. Beneath this zone, tubular invasion of bacteria is frequently seen. The bacteria invasion has as consequence the pulp inflammation, which may have serious consequences as pain, pulpar necrosis and periapical lesions^7^^,^^16^.

### Hypersensitivity

Dentine hypersensitivity has been defined as a sharp, short pain arising from exposed dentin in response to stimuli typically thermal, evaporative tactile, osmotic, chemical and which cannot be ascribed to any other form of dental defect or pathology^31^. The short and sharp pain symptoms are thought to be derived from the hydrodynamic challenge.

The most affected patients range from 20 to 40 years-old; premolars and incisors tend to be most sensitive teeth, being the pain localized on the facial surface. Sensitive teeth have much greater numbers of open tubules per unit area and the average diameter of tubules is almost 2 times greater than tubules in nonsensitive teeth^32^.

Dentine hypersensitivity represents a condition of presumable multifactorial pathology. Two processes are essential for its development: (1) dentin must be exposed through genetic disturbance, enamel defect (lamellae and spindles), loss of enamel (erosion, abrasion, attrition, abfraction), gingival recession with rapid loss of cementum and (2) the dentin tubules must be open to both the oral cavity and the pulp.

Diagnostic protocol for this condition consisted of Medical, Dental Dietary, Oral Hygiene History and Intra-oral examinations with air indexing method. The treatments in the office can be made with substances that are able to create a smear-layer on dentin surface, occluding dentinal tubules with insoluble precipitates and stimulating the production of reparative dentin and/or sclerotic. This can be achieved chemically with agents like potassium, calcium and fluoride or physically^32^^,^^33^.

### Clinical impact of the knowledge

Based on above considerations, medical and dentist teams need to consider potential dental problems after bariatric surgery, and to supply their patients with the appropriate information and instructions regarding oral hygiene maintenance, healthy dietary patterns and regular dental health monitoring by a dentist or dental hygienist.

## CONCLUSION

The present review suggests that postsurgical meal patterns and gastric reflux might increase the risk for dental lesions, particularly in the presence of other risk factors such as consumption of sweet-tasting foods and acidic beverages. However, due to a lack of data, more research is needed to evaluate this relationship.
